# Preservation of Differentiation and Clonogenic Potential of Human Hematopoietic Stem and Progenitor Cells during Lyophilization and Ambient Storage

**DOI:** 10.1371/journal.pone.0012518

**Published:** 2010-09-01

**Authors:** Sandhya S. Buchanan, David W. Pyatt, John F. Carpenter

**Affiliations:** 1 Center for Pharmaceutical Biotechnology and Department of Pharmaceutical Sciences, University of Colorado, Aurora, Colorado, United States of America; 2 Summit Toxicology, Superior, Colorado, United States of America; University of Minho, Portugal

## Abstract

Progenitor cell therapies show great promise, but their potential for clinical applications requires improved storage and transportation. Desiccated cells stored at ambient temperature would provide economic and practical advantages over approaches employing cell freezing and subzero temperature storage. The objectives of this study were to assess a method for loading the stabilizing sugar, trehalose, into hematopoietic stem and progenitor cells (HPC) and to evaluate the effects of subsequent freeze-drying and storage at ambient temperature on differentiation and clonogenic potential. HPC were isolated from human umbilical cord blood and loaded with trehalose using an endogenous cell surface receptor, termed P2Z. Solution containing trehalose-loaded HPC was placed into vials, which were transferred to a tray freeze-dryer and removed during each step of the freeze-drying process to assess differentiation and clonogenic potential. Control groups for these experiments were freshly isolated HPC. Control cells formed 1450±230 CFU-GM, 430±140 BFU-E, and 50±40 CFU-GEMM per 50 µL. Compared to the values for the control cells, there was no statistical difference observed for cells removed at the end of the freezing step or at the end of primary drying. There was a gradual decrease in the number of CFU-GM and BFU-E for cells removed at different temperatures during secondary drying; however, there were no significant differences in the number of CFU-GEMM. To determine storage stability of lyophilized HPC, cells were stored for 4 weeks at 25°C in the dark. Cells reconstituted immediately after lyophilization produced 580±90 CFU-GM (∼40%, relative to unprocessed controls p<0.0001), 170±70 BFU-E (∼40%, p<0.0001), and 41±22 CFU-GEMM (∼82%, p = 0.4171), and cells reconstituted after 28 days at room temperature produced 513±170 CFU-GM (∼35%, relative to unprocessed controls, p<0.0001), 112±68 BFU-E (∼26%, p<0.0001), and 36±17 CFU-GEMM (∼82%, p = 0.2164) These studies are the first to document high level retention of CFU-GEMM following lyophilization and storage for 4 weeks at 25°C. This type of flexible storage stability would potentially permit the ability to ship and store HPC without the need for refrigeration.

## Introduction

Hematopoietic stem and progenitor cells (HPC) contain populations of cells with long-term and short-term regeneration capacities as well as committed progenitors. HPC give rise to all the blood cell types including lymphoid (T-cells, B-cells, NK-cells, dendritic) and myeloid (monocyctes/macrophages, neutrophils, megakaryocytes, granulocytes, eosinophils, erythrocytes)[1]. These cells can be isolated from bone marrow, peripheral blood, or human umbilical cord blood (UCB) and have been shown to be valuable for treatment of a wide variety of human diseases[2], [3], [4], [5], [6], [7], [8]. As a result, there is a growing need for producing, storing and shipping large numbers of HPC to ensure a steady supply for use in clinical applications. Simple preservation techniques, such as refrigeration or tissue culture, have drawbacks including limited shelf life, high cost and risk of contamination. Alternatively, cryopreservation has been successfully utilized for long-term storage of HPC. This approach is based on the principle that chemical, biological and physical processes are sufficiently “suspended” at cryogenic temperatures (−196°C) because liquid water does not exist below −130°C[9]. In fact, the only physical states that exist at cryogenic temperature are crystalline or glassy (also called amorphous), and in both the viscosity is so high (≥10^13^ poise) that the rates of diffusion controlled reactions are insignificant on practical time scales[10], [11], [12]. Furthermore, there is insufficient thermal energy for chemical and metabolic processes to proceed at practical relevant rates[9], [13].

Conventionally, cryopreservation protocols employ high relative concentrations (e.g., 1.0–2.0 M) of cryoprotectants such as dimethyl sulfoxide (DMSO), glycerol or ethylene glycol[9], [14] because they readily penetrate cell membranes and thus confer protection to the intracellular components. Some complications associated with the use of these permeable cryoprotectants include an exceptionally low glass transition temperature (<−83°C)[15]. Low glass transition temperatures require tight control of temperature during product storage and transfer. Thermal excursions or storage above the glass transition temperature results in an increase in motion as a result of the large decrease in matrix viscosity. Decreased viscosity allows diffusion to occur and can result in degradation of frozen cells and tissues[9], [16], [17], [18].

More importantly, freezing media containing DMSO have shown to be toxic to patients[19], [20], [21], [22], [23]. Efforts to reduce toxicity include the removal of DMSO prior to transfusion or decreasing the amounts used in the freezing process[24], [25], [26]. An alternative to DMSO includes nontoxic sugars such as trehalose. Trehalose is a non-toxic, non-reducing disaccharide of glucose that possesses an exceptional ability to stabilize and preserve cells and cellular structures during freezing[14], [27], [28], [29], [30] and drying[31], [32]. The utility of trehalose as a cryoprotectant has been attributed to its protective interactions with lipid membranes, stabilization of labile proteins during freezing-thawing, its chemical stability and the ability to form stable glasses during cooling of cells for cryopreservation[31], [32], [33], [34], [35], [36], [37]. For cell preservation, formation of a glassy matrix in the cell's interior also can contribute to the inhibition of potentially lethal intracellular ice crystals and minimize cell damage to excessive concentration of electrolytes. One obstacle in the utility of trehalose as a cryoprotectant is overcoming its impermeability to mammalian cell membranes, which has been investigated extensively[31], [38], [39], [40], [41], [42], [43], [44], [45], [46], [47].

As a first step toward preserving HPC by freeze drying, we previously investigated the permeabilization of a model cell line (TF-1) to trehalose[48]. TF-1 cells are CD34 positive, cytokine-dependent, and retain the ability to differentiate into erythroid, monocytic and megakaryocytic lineages following appropriate stimulation[49], [50], [51], [52], [53]. These characteristics make them an appropriate surrogate for primary HPC in evaluating the effects of the critical processing steps. TF-1 cells were permeabilized to trehalose using an endogenous cell surface receptor, termed P2Z[54], [55], [56], which is synonymous with P2X_7_ [57]. In the presence of millimolar concentrations of extracellular uncomplexed adenosine-5-triphosphate (e.g., ATP^4−^), this protein forms nonselective pores that render plasma membranes permeable to small molecules (e.g., MW≤900 Da)[58], [59], [60]. This effect is reversed by a ten-fold dilution (to reduce the ATP concentration) and the addition of magnesium to complex with ATP, so that cells can be permeabilized transiently[61]. The average number of TF-1cells positive for the P2Z receptor was approximately 86%. Cells loaded with 200 mM (6.8% w/v) trehalose were frozen and stored at −80°C for 4 months. Following storage, thawed cells were evaluated for differentiation capacity and clonogenic output. Results obtained with this technique were compared to conventional freezing protocols using 10% (v/v, 1.4 mM) dimethyl sulfoxide (DMSO). Colony forming units (CFU) generated from cells frozen with intra- and extracellular trehalose were essentially equivalent in size, morphology, and number (∼91%) to those generated from unfrozen cells. Furthermore, there were no significant alterations in phenotypic markers of differentiation (CD34, CD33, and CD235a), activation (CD38), and proliferation (CD71). The P2Z receptor is expressed in many hematopoietic cells including myeloid progenitors, CD34^+^ peripheral blood progenitor cells as well as neutrophils, dendritic, granulocytes, monocytes/macrophages, lymphocytes, and erythrocytes[58], [62], [63], [64].

In this study we explore the ability of intra- and extracellular trehalose to protect human HPC during freezing, drying and storage at room temperature for 4 weeks. To ensure maximum uptake of sugar, cells were permeabilized via the P2Z receptor with two different types of porating agent: ATP and benzoyl ATP (BzATP), a more potent agonist. Successful preservation criteria for HPC include retaining the ability to form colonies in semi-solid culture medium and the capability of differentiating to multiple lineages. More mature progenitor cells are capable of forming colong forming unit-erythroid (CFU-E), burst forming units-erythroid (BFU-E), colony forming unit-granulocyte (CFU-G), megakaryocyte (CFU-Mk), and colony forming unit-granulocyte monocyte/macrophage (CFU-GM), a heterogeneous population of macrophages and granulocytes. Colonies comprised of granulocyte-erythroid-megakaryocyte-macrophage (CFU-GEMM) represent a primitive multi-lineage myeloid progenitor cell that are present in UCB in relatively high numbers compared to more mature cells[1], [65], [66]. The number of CFU observed can vary depending on the frequency of individual lineage-specific progenitor cells present in UBC obtained from individual subjects and the specific culture conditions. In addition, variability in the number of CFU may occur because we did not quantify nor isolate and identify the types of cells that were positive for the P2Z receptor.

To maximize cell survival during freeze-drying and subsequent storage, it also is important to obtain an optimal residual water content and a sufficiently high glass transition temperature (Tg) to permit long-term storage ambient storage. Therefore, we also investigated the effects of lyophilization processing steps and resulting residual water content on both cell survival and Tg.

## Materials and Methods

### Reagents

Sodium bicarbonate, ammonium chloride, sodium ethylenediaminetetracetic acid (EDTA), 2,3-(4-benzoyl) benzoyl adenosine-5-triphosphate (BzATP) and adenosine-5-triphosphate were purchased from Sigma (St. Louis, MO). Trypan blue, phosphate buffered saline (PBS) without calcium and magnesium, fetal bovine serum (FBS) and Lymphocyte Separation Medium (density = 1.077 g/mL) were purchased from Fisher Scientific (Atlanta, GA). MethoCult® Classic was purchased from Stem Cell Technologies (Seattle, WA). Hydroxyethyl starch (Viastarch, HES) was purchased from Fresenius (Linz, Austria). The HES mean molecular weight, water content, and NaCl content were 259,700 Da, 4.64% and 0.11%, respectively[67]. Bovine serum albumin fraction-V 30% (BSA) was purchased from Gemini BioProducts (West Sacramento, CA). Low endotoxin glucose and trehalose were purchased from Pfanstiehl Laboratories (Waukegan, Illinois). Fluorescein-5-isothiocyanate isomer I (FITC) and propidium iodide (PI) was purchased from Molecular Probes (Eugene, OR).

### Red blood cell lysis buffer

8.3 g ammonium chloride, 1.0 g sodium bicarbonate, and 0.04 g sodium EDTA were dissolved in 1 L ultra pure sterile water. The solution was filter through 0.22 µM filter and stored at 4°C. Red blood cell (RBC) lysis buffer was warmed to 37°C before use.

### Isolation HPC from human umbilical cord blood

All protocols were approved by the University of Colorado Health Sciences Center Internal Review Board (number 01-809). After informed consent, human cord bloods were obtained using sterile tubes with heparin from newborn umbilical cords following normal vaginal deliveries. Patient consent was written. A translator was available if required or a copy in the native language of the patient was made available as requested. Only UCB that tested negative for HIV were used in this study. Samples were processed within 12 hr of collection.

Cord blood specimens were diluted with an equal volume of RBC lysis buffer and transferred to a 50 mL conical tube. Each specimen tube was then rinsed with 2 mL of RBC lysis buffer and the rinse was added to specimens. Specimens were briefly vortexed and then incubated for 3 minutes at room temperature. Cells were pelleted by centrifugation at 1500 rpm for 10 minutes. The supernatant was decanted and cells were resuspended in PBS/1%BSA equivalent the original specimen volume.

Light-density mononuclear cells (MNC) were isolated by layering cell suspension carefully onto Lymphocyte Separation Medium (density = 1.077 g/mL, 2 volumes of diluted blood to 1 volume of separation medium) and centrifuging at 1500 rpm for 30 minutes at room temperature. MNC (monocytes and lymphocytes) have a lower buoyant density than the erythrocytes and the polymorphonuclear (PMN) leukocytes (granulocytes). Since the majority of MNC have densities below 1.077 g/mL, they can be isolated at the sample/medium interface. The interphase layer containing MNC was collected and washed twice with PBS/1%BSA. After 2 cycles of washing and centrifugation, cell pellets were resuspended in 5 mL of PBS/1%BSA. Cells were counted using a Coulter Counter, and cell viability was assessed by Trypan blue exclusion. No further cell identification or purification was performed.

MNC cells were cultured with MethoCult® Classic. Cytokines in this medium only support cells that give rise to CFU-E, BFU-E, CFU-GM, and CFU-GEMM. All references to these cells will be identified as HPC in the text. (See CFU assay for details)

### Expression of P2Z Receptor on mononuclear cells

Expression of P2Z receptor was measured by permeabilizing HPC with BD Cytofix/Cytoperm kit (BD Biosciences Pharmingen, San Diego, CA) using manufacturer's instructions, staining with anti-P2Z/P2X7 receptor (Calbiochem, La Jolla, CA) polyclonal antibody from rabbit and anti-Rabbit-PE secondary antibody developed in goat (Sigma, St. Louis, MO). Briefly, 1×10^6^ cells were washed twice with 1 mL of staining buffer (PBS/1% FBS/5% (w/v) sodium azide). To fix cells, HPC were pelleted by centrifugation (1100 rpm for 8 minutes) and then resuspended in 250 µL of BD Cytofix/Cytoperm solution for 30 minutes at 4°C. HPC were washed twice in 1 ml of 1X BD Perm/Wash solution. Cells were then resuspended in 50 µL of BD Perm/Wash solution containing anti-P2Z/P2X7 (1∶100) for 30 minutes at 4°C. Cells were washed twice in 1 mL of 1X BD Perm/Wash solution. Cells were then resuspended in 50 µL of 1X BD Perm/Wash solution containing 20 µL of anti-Rabbit-PE for 30 minutes at 4°C in the dark. Cells were washed twice in 1 mL staining buffer. Cells were then resuspended in 300 µL of staining buffer and stored at 4°C in the dark until flow cytometric analysis. Flow cytometric analysis was performed a Coulter Epics XL equipped with Summit v3.1 and v4 software.

### Cell poration and trehalose loading

HPC were incubated in poration buffer containing 100 µM BzATP or 5 mM ATP and 200 mM trehalose at a concentration of 1×10^6^ cells in 1 mL. Poration buffer is composed of 5 mM glucose, 1X essential amino acids, 1X nonessential amino acids and 1X Vitamin Stock in 10 mM K_2_HPO_4_/KH_2_PO^4^ titrated to pH 7.45±0.03 (modified from Menze[68]. Cells were incubated in the reaction mixture for 0–90 min to accommodate trehalose loading. Pores were closed with the addition of 1 mM MgSO_4_ and 10-fold dilution of BzATP or ATP using RPMI 1640.

Poration efficiency for HPC was assessed by FITC/PI staining using flow cytometry as previously described[48], [69], [70], [71], [72]. A stock solution of FITC for permeabilization studies was prepared by dissolving 1 mg FITC in 1 mL of DMSO, with further dilution to 0.1 mg/mL using PBS without calcium and magnesium. Briefly, porated cells were loaded with trehalose containing 100 µL of 0.1 mg/mL FITC. Pores were closed and excess dye was removed by washing twice with PBS/1%FBS. Approximately 1×10^5^ porated cells were removed and suspended in 500 µL of PBS/1%FBS. Cells were treated with 10 µL of 1.0 mg/mL PI, a membrane-impermeant dye, for 4 min prior to analysis. PI labels the nucleic acids of membrane-compromised cells. Cells which stained positive for FITC and negative for PI were gated and considered to be both viable and porated, whereas cells stained with PI were gated and considered to be dead. All reported values were normalized to total cell count. Flow cytometric analysis was performed on a Coulter Epics XL equipped with System II software.

### Lyophilization

After trehalose loading, HPC were lyophilized in a formulation containing 6.8% trehalose/2% HES/5% HSA (w/v). Approximately 1 mL of sample solution was transferred to 5-mL flat bottom borosilicate lyophilization vials (West Pharmaceutical, Lititz, PA), and the vials were placed on the shelf of an FTS DuraStop MP/DuraDry MP freeze-dryer (Stone Ridge, NY). The shelf was cooled from room temperature to −45°C at a cooling rate of 2.5°C/min until product temperature reached −45°C. Samples were maintained −45°C for 180 minutes. Primary drying was initiated by raising the shelf temperature from −45°C to −35°C at 2.5°C/ minute, reducing the chamber pressure to 80 mTorr and maintaining the shelf temperature at −35°C for 2160 minutes. To determine completion of primary drying, the step was monitored using the chamber pressure rise test and equilibration of the product temperature to the shelf temperature, as described previously[69], [70]. For secondary drying, samples were warmed by increasing the shelf temperature at a rate of 0.2°C/minute to the designated secondary drying temperature to be tested and maintaining the shelf at this temperature for 360 minutes. To determine where clonogenic and differentiation potential is lost during lyophilization, vials were removed at the end of primary drying (−35°C) and during secondary drying (at 5°, 10°, 15°, and 20°C) from the freeze-drying chamber into an antechamber using a retractable arm. The extracted vials were stoppered in the antechamber under the same pressure as the chamber (∼80 mTorr). At the end of the cycle, the trays were raised until the rubber stoppers were pushed down to seal the vials under vacuum before removing them from the unit. All samples were stored at −80°C until analysis.

### Rehydration

Lyophilized samples were rapidly reconstituted using high purity water. An aliquot of ∼950 µL water was drawn into a 1 mL serological pipette, and the water was released down the sides of lyophilization vials. To ensure homogeneous resuspension of the cells, the solution containing the cells was then gently drawn into a 1 mL serological pipette and released down the sides of the vials 5-times. Approximately 2 mL of complete medium was added to each vial, and cells were transferred to 10 cm^2^ flat bottom tube flasks (MidWest Scientific, St. Louis, MO), and incubated for 2 hr at 37°C in humidified air containing 5% CO_2_ before plating cells in methylcellulose.

### Colony Forming Unit Assay

Progenitor cell proliferation of control, frozen and thawed or freeze dried, and rehydrated cells was quantified by an *in vitro* method using MethoCult® Classic, a semi-solid methylcellulose system containing stem cell factor (SCF), GM-CSF, interleukin 3 (IL-3), and erythropoietin (StemCell Technologies, Vancouver, BC, Canada) and by measuring clonogenic activity via CFU. The assay was performed with light-density MNC as described above. Cytokines in this medium only support cells that give rise to CFU-E, BFU-E, CFU-GM, and CFU-GEMM. The culture plates used in these studies do not support the growth of anchorage-dependent cells. All references to these cells will be identified as HPC in this text.

Unfrozen control cells were suspended at a concentration of 1×10^6^ cells/mL and 100 µL of cells were removed for culture in 1% methylcellulose. (∼1×10^5^ as determined by both a Coulter counter and trypan blue exclusion). The 100 µL of cells was added to 5 mL of 1% methylcellulose (∼20,000 cells/mL). The clonogenic activity of unfrozen control cells was standardized in this manner and considered to be 100% clonogenic potential. Colony identification was based on color and morphology. Only multi-lineage myeloid colonies were scored (i.e., CFU-GEMM, CFU-GM, or BFU-E).

For freeze-thawing experiments, cell samples were removed at the end of the freezing step of lyophilization and stored at −80°C until analysis. Frozen samples were rapidly thawed in a 37°C water bath. For experiments of cells that were freeze-dried, samples were stored at −80°C until analysis. Dried samples were rehydrated as described in the rehydration section above. Following thawing or rehydration, approximately 2 mL of complete medium was added to each sample. Cells were mixed by pipetting with a 5-mL serological pipette 5-times to ensure homogeneous suspension. Cells were then transferred to 10 cm^2^ flat bottom tube flasks (MidWest Scientific, St. Louis, MO) and incubated for 2 hr at 37°C in humidified air containing 5% CO_2_. Following incubation at 37°C, approximately 50 µL of cell suspension was transferred to a 15-mL conical tube with 6 mL of 1% methylcellulose containing growth factors.

Four individual culture plates were scored for each group. All samples were cultured at 37°C in humidified air containing 5% CO_2_ for 14 days, after which colonies were enumerated on an inverted Nikon microscope (1 colony ≥20 cells).

### T_g_ measurements

All samples were analyzed on a Perkin-Elmer Diamond DSC equipped with an Intercooler II and the data were analyzed on Pyris 7.0 software. The DSC was calibrated using indium (for temperature and enthalpy of melting). Vials containing dried samples used for T_g_ measurements were placed in a nitrogen-purged dry glove box to avoid possible hydration resulting from exposure to environment and hermetically sealed in aluminum pans. Approximately 10 mg of solid was measured each measurement. Lyophilized samples were heated from 0 to 200°C at100°C/minute, maintained at 200°C for 5 minutes, then cooled to 0°C and reheated again to 250°C at the same rates to measure T_g_ in the second scan. Values are reported as the onset temperature of the glass transition.

### Residual water content

Residual water content of lyophilized samples was determined by Karl Fischer titration using a Mettler DL37 coulometric moisture analyzer (Hightstown, NJ) containing pyridine free vessel solutions (Photovolt Instruments, Inc., St. Louis Park, MN). Samples were prepared in a nitrogen-purged dry glove box to avoid possible hydration resulting from exposure to environment. Lyophilized samples were dissolved in 1 mL of dimethyl formamide. The samples were sonicated for 15–25 minutes to ensure complete solubility prior to analysis. Using a gas-tight syringe, 250 µL were withdrawn through the rubber stopper and moisture analysis was performed as previously described^70^. Values are reported as mass percent water (gH_2_O/100 g solid).

### Storage stability

After lyophilization, stoppered vials containing dehydrated samples were sealed with aluminum caps and stored in an incubator at 25°C (so-called “controlled room temperature”) in the dark for 4 weeks. At the end of the storage period, samples were rehydrated and analyzed as described above.

### Statistical Analysis

Statistical analyses were performed on CFU assay results using paired-student t test (SigmaPlot 2001) and ANOVA using Dennett's test, which controls overall error rate for comparing all other means with a designated control group's mean (JMP v6-8). Differences among groups were considered significant when *p* value was less than 0.05. Data reported as the mean ± the standard deviation of three independent experiments each with quadruplicate samples.

## Results

### Expression of P2ZR and permeabilization of HPC

**Figure 1:**
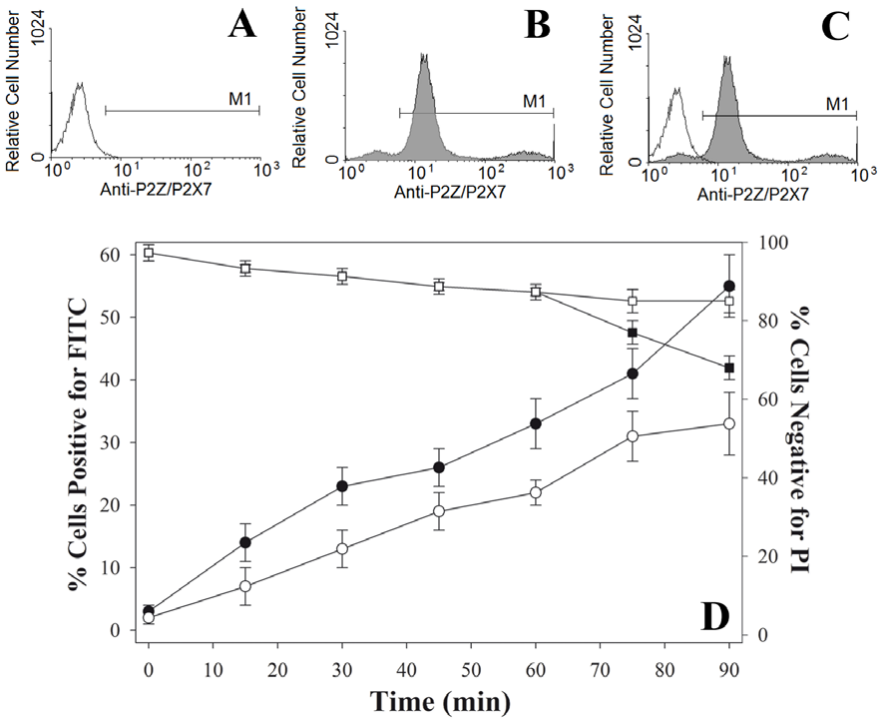
Expression of P2Z receptor and dye uptake by porated HPC. (A) Expression of P2Z receptor was measured by immunofluorescence and analyzed by flow cytometry. Negative control treated with secondary antibody alone signifying non-specific binding or background fluorescence (approximately 1.6±0.3%). (B) Cells stained with anti-P2Z/P2X7-PE showed approximately 58±4% of HPC cells express the P2Z receptor compared to negative control. (C) Overlay of panels A and B showing comparison of background fluorescence to cells positive for P2Z. D) Poration efficiency of HPC: cells permeabilized in the presence of 200 mM trehalose for 0–90 min using 5 mM ATP (Open Circles) and 100 µM BzATP (Closed Circles). Poration efficiency was quantified by determining FTIC-positive cells with flow cytometry. Membrane integrity following permeabilization using 5 mM ATP (Open Squares) and 100 µM BzATP (Closed Squares) was measured by quantifying propidium iodide-negative cells with flow cytometry.

We investigated the expression of P2Z receptor on HPC using indirect staining and flow cytometric analysis (Figure 1). Staining with secondary antibody alone marked the background fluorescence level by non-specific binding of this antibody to dead or other cells (1.6%±0.3, Figure 1A). Fluorescence levels above the background were considered specific for P2Z and shown to be approximately 58%±4% (Figure 1B) compared to secondary antibody alone (Overlay of A and B as shown in Figure 1C).

To assess poration efficiency, HPC were permeabilized by exposing them to either 5 mM ATP or 100 µM BzATP, which opens the P2Z cell surface receptor, in medium containing 200 mM trehalose and 0.1 mg/mL FITC. FITC was employed as an indicator of poration because it is normally impermeable to cellular membranes and has a similar molecular mass (MW 389) to that of trehalose (MW 342). In the absence of porating agent, less than 1% of cells were positive for the presence of dye. There was a gradual increase in the number of cells positive dye when permeabilized in the presence of ATP, reaching a maximum of ∼31% after 75 minutes (Figure 1D). Permeabilization in the presence of BzATP (a more potent agonist for the P2Z receptor than ATP)[57], [60], resulted in a steady increase in the number of cells positive for dye, with 58±4% of cells positive for dye after 90 minutes. The membrane integrity of cells was also monitored during poration using PI. There was a gradual loss in membrane integrity after 90 minutes of permeabilization using BzATP as the porating agent, with 68±3% of cells remaining intact whereas 90±4.5% of cells remained intact when the porating agent was ATP. These results indicate that HPC can be permeabilized via P2Z receptors for up to 60 minutes using BzATP and for up to 90 minutes using ATP without a significant decrease in cell viability.

### Effect of intracellular trehalose on the frequency of CFU-GM, BFU-E, and CFU-GEMM following freeze-thawing

**Figure 2:**
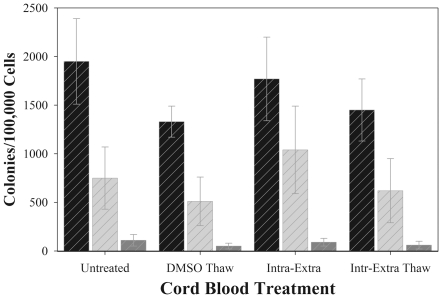
HPC cell proliferation and differentiation of untreated and cryopreserved cells. Frequency of CFU-GM (Black Stripes), BFU-E, (Light Gray Stripes) and CFU-GEMM (Dark Gray Stripes) colonies following cryopreservation with trehalose were quantified using CFU assay (see Materials and Methods). Values are represented as the mean ± standard deviation of the mean of 3 independent experiments for *n* = 3 subjects with 4 replicates and 4 individual culture plates for each control or treatment group (1 colony = ≥20 cells).

Cells must first survive freezing before freeze-drying can be attempted. To demonstrate the use of trehalose as an appropriate intracellular cryoprotectant, we permeabilized cells in medium containing 100 µM BzATP and 200 mM trehalose for 75 minutes. Trehalose loaded cells were suspended in a formulation containing 6.8% trehalose/2% HES/5%HSA and then slowly cooled (1°C/minute) to −80°C followed by storage at −80°C for one week. Cryopreserved cells were rapidly thawed in a 37°C water bath and tested for clonogenic potential. Control groups for these experiments were freshly isolated cells and cells cryopreserved with 10% DMSO (1.4 M). As seen in Figure 2, cells cryopreserved trehalose produced comparable CFU to cells cryopreserved with DMSO. These data document that primary HPC loaded with trehalose can survive freeze-thawing when cryopreserved in medium containing 6.8% extracellular trehalose/2% HES/5% HSA. Furthermore, the frequency of CFU-GM, BFU-E, and CFU-GEMM is comparable to results obtained with fresh cells and those cryopreserved with DMSO.

### Effect of lyophilization and reconstitution on frequency of CFU-GM, BFU-E, and CFU-GEMM

**Figure 3:**
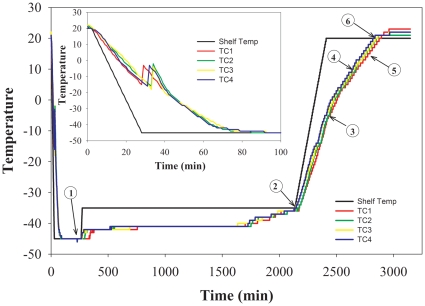
Representative plot of processing variables. Graph representing processing variables monitored during lyophilization used to optimize processing conditions. Optimization conditions were determined by testing vials removed during freeze drying cycle. Sample extraction was performed with a retractable arm via an antechamber equipped with an independent vacuum pump so samples could be removed at the following shelf temperatures: (1) end of freezing step at −45°C, (2) end of primary drying at −35°C, (3) 5°C during secondary drying, (4) 10°C during secondary drying, (5) 15°C during secondary drying, and (6) 20°C during secondary drying. Chamber was at ambient temperature and atmospheric pressure during the freezing step, and approximately 10°C and 80 mTorr during primary and secondary drying.

**Figure 4:**
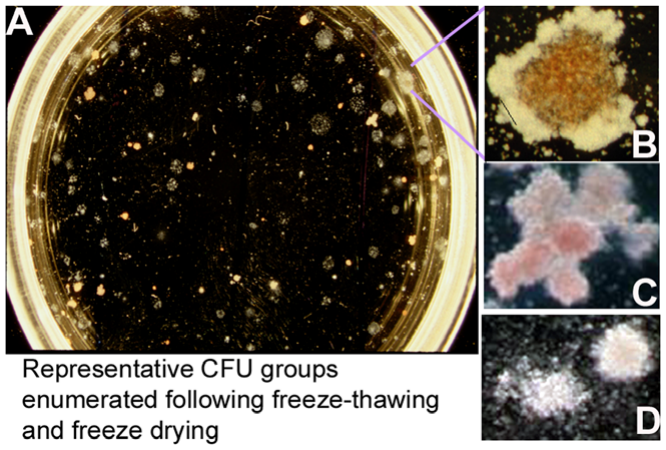
Representative images of colony forming unit assay plate and colony scoring. Appearance of: A) Semi-solid methylcellulose containing growth factors (SCF, IL-3, and EPO). B) Colonies scored as CFU-GEMM, C) Colonies scored as BFU-E, and D) Colonies scored as CFU-GM.

**Figure 5:**
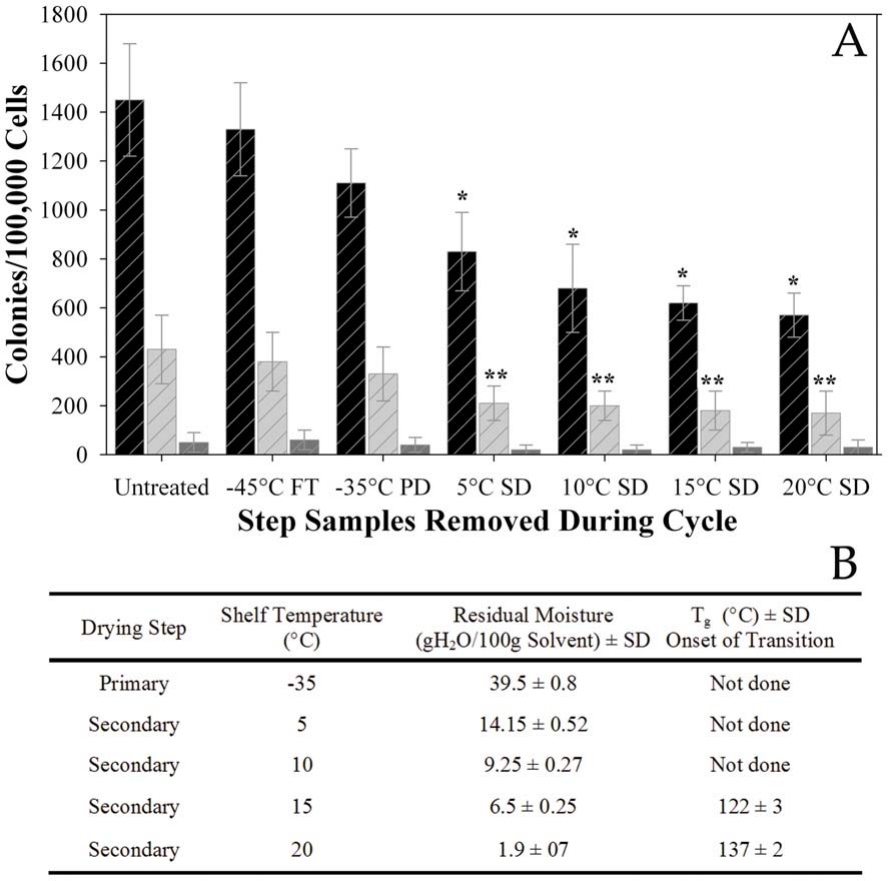
Frequency of CFU-GM, BFU-E and CFU-GEMM colonies obtained following lyophilization and reconstitution. A) HPC proliferation and differentiation of untreated and lyophilized cells were quantified by CFU assay (see Materials and Methods). Colonies were scored as CFU-GM (Black Stripes), BFU-E (Light Gray Stripes), or CFU-GEMM (Dark Gray Stripes). Values are represented as the mean ± standard deviation of the mean (p<0.001) of 3 independent experiments for *n* = 3 subjects with 4 replicates and 4 individual culture plates for each control or treatment group. * = statistically different from control and other test groups, ** = statistically different from control, but not statistically different from each other. B) Thermal analysis of samples removed during lyophilization. FS = freezing step, PD = primary drying, SD = secondary drying.

A representative plot of the temperature profile during the freeze drying cycle shows when vials were removed during lyophilization without interrupting the cycle to assess the effect of each step on the differentiation and clonogenic potential of the cells (Figure 3). Trehalose loaded cells were lyophilized in a formulation containing 6.8% trehalose/2% HES/5% HSA (w/v). Samples were removed during processing using an extractable arm into an antechamber (See methods) and stored at −80°C until analysis. There were three main types of colonies that were enumerated: CFU-GM, BFU-E, and CFU-GEMM (Figure 4). There was no difference in the number of CFU produced by control groups and cells that were removed at the end of the freezing step and thawed (Figure 5A). There was a gradual decrease in the number of CFU-GM and BFU-E for samples removed at the end of primary drying and during secondary drying; however, there were no differences in the number of CFU-GEMM (∼82%). An ANOVA was performed on the results and the means were compared using Dennett's test, which controls overall error rate for comparing all other means with a designated control group's mean. CFU produced by thawed cells removed at the end of the freezing step and rehydrated cells removed at the end of primary drying were not statistically different from that for control cells. There was a gradual decrease in the number of CFU-GM and BFU-E produced by cells removed at increasing temperatures during secondary drying, which were statistically different from control groups. In marked contrast, cells produced approximately 82% CFU-GEMM at all secondary drying shelf temperatures compared to control cells. There were no statistical differences in the means between CFU-GEMM produced by rehydrated cells and control cells.

In addition to measuring the differentiation and clonogenic potential of lyophilized cells, we also quantified the Tg and residual water content of the dry cake (Figure 5B). Not surprisingly, the Tg of samples increased as water was removed from samples. The final Tg of samples processed at a secondary drying temperature of 20°C was 197±3°C.

At the end of the primary drying step, there was approximately 39.5±0.8 gH_2_O/100 g solid. As expected, the residual water content decreased as secondary drying continued, which resulted in a concomitant decrease in cells' differentiation and clonogenic potential. This observation is consistent with results reported by Toner and colleagues who suggest that there may be an optimal residual water content required for stable dry cells[31].

### Effect of storage for 4 weeks at 25°C on frequency of CFU-GM, BFU-E, and CFU-GEMM

To determine storage stability of lyophilized HPC, cells dried at a final secondary drying shelf temperature of 15°C were stored for 4 weeks at 25°C in the dark (Table 1). Cells reconstituted immediately after lyophilization produced approximately 40% CFU-GM, 40% BFU-E, and ∼82% CFU-GEMM relative to control. Cells reconstituted on after 28 days of storage at room temperature produced approximately 35% CFU-GM 26% BFU-E, and 82% CFU-GEMM compared to control. There was an initial decrease in the frequency of CFU-GM and BFU-E upon reconstitution immediately after lyophilization, but there was no further change in clonogenic output after 4 weeks of storage. There was not a significant difference in the frequency of CFU-GEMM by cells stored for 4 weeks at 25°C and untreated control cells. These results indicate that the initial loss observed during secondary drying is the main loss of cells during freeze-drying and storage in the lyophilized formulation.

**Table 1:** Storage stability of lyophilized HPC derived from umbilical cord as a function of clonogenic activity.

CFU Type	Untreated control	CFU Colonies/50 µL ± SD immediately after reconstitution	CFU Colonies/50 µL ± SD Storage @ 25°C for 4 wks in the dark
CFU-GM	1450±230	570±90 (∼40%)	510±170 CFU-GM (∼35%)
BFU-E	430±140	170±70 BFU-E (∼40%)	112±68 BFU-E (∼26%)
CFU-GEMM	50±40	41±22 CFU-GEMM (∼82%)	36±17 CFU-GEMM (∼82%)

HPC were lyophilized in a formulation containing 6.8% trehalose/2% HES/5% HSA (w/v) and stored at 25°C for 4 weeks in the dark. Stability of CFU-GM, BFU-E, and CFU-GEMM.

## Discussion

Requirements for successful stabilization of cells during freezing and drying include appropriate cryoprotectants and lyoprotectants to preserve cellular components and the dependent functions. Endogenous pore forming proteins, such as the P2Z receptor, are ideal for transport of low molecular weight compounds. P2Z receptors are activated specifically by the fully ionized form of ATP (e.g., ATP^4−^). The pore opening is also temperature and pH dependent. Optimal conditions for pore formation is 37°C and pH 8.0–8.5, whereas the pore is inactive below 18°C and at pH 6.5[73]. To reduce cellular stress during permeabilization, we loaded trehalose into cells at 37°C in buffer with a pH of 7.45. The fraction of cells positive for the P2Z receptor was approximately 58%, which indicates that not all HPC derived from UBC express this pore forming protein. We did not see differences in the differentiation and clonogenic potential of trehalose-loaded HPC after freezing and thawing compared to control groups. However, we did observe a significant difference in differentiation and clonogenic potential after rehydration of desiccated cells capable of producing BFU-E and CFU-GM. The non-ubiquitous expression of the P2Z receptor may explain the differences observed in the number of colonies produced by cells before and after freeze-drying.

To identify at point during during freeze-drying that cells lost function, we removed samples during the various steps of the lyophilization process. Lyophilization is a dehydration process comprising three main steps: freezing, primary drying, and secondary drying. The purpose of freezing is to separate the solvent (e.g., water) from the solutes. In a typical aqueous solution, the a large fraction of water will form ice crystals and the solutes will be concentrated in the remaining solution between the ice crystals. As cooling continues, the concentrated non-ice phase solidifies; yielding an amorphous matrix.

Upon completion of freezing, the lyophilization cycle moves into the primary drying phase. The main purpose of primary drying is to remove the water from the formulation by subliming the ice crystals formed during freezing. Primary drying begins by reducing the chamber pressure below the vapor pressure of ice and raising the shelf temperature to supply heat for sublimation of ice. The ice-gas interface begins at the top of the cake and proceeds downwards to the bottom of the vial. Upon completion of primary drying, there is still a considerable amount of residual water remaining in the formulation.

The purpose of secondary drying is to reduce to remove the water that did not freeze and in the amorphous phase by desorption, without compromising the volume of the cake formed by the partially-dried formulation. Desorption of water is accomplished by raising the shelf temperature. Secondary drying is complete when the desired residual water content is achieved for long-term stability (product and formulation specific).

During the first two steps of lyophilization, there was not a significant reduction in differentiation and clonogenic potential of HPC loaded with trehalose. When samples were removed during secondary drying, only cells capable of producing CFU-GEMM survived to levels comparable to control groups. ANOVA performed on results suggests the critical processing step for BFU-E reduction is during secondary drying at 5°C. CFU produced by cells exposed to higher secondary drying temperatures were not statistically different from those removed from the lyophilizer after secondary drying at 5°C. It may be possible to increase differentiation and clonogenic potential by simply optimizing the freeze-drying process. The number of CFU-GM produced by cells removed at 5°C were also significantly different from control groups. However, there was a gradual decrease in the number of CFU-GM produced by cells removed downstream from this time point, suggesting the damage may have occurred at the onset of secondary drying which result in cumulative losses in CFU production. These cells may require both cycle optimization and additional formulation development to increase clonogenic potential to levels obtained with CFU-GEMM. It is unknown if CFU-GEMM express a higher number of P2Z receptors, and as a result, have an increased survival rate due to higher amounts of intracellular trehalose.

The frequency of BFU-E, CFU-GM, and CFU-GEMM observed in this study are significantly greater than results reported previously by Natan and colleagues[74]. In their study, human UBC were preserved with the antioxidant epigallocatechin gallate (EGCG) and trehalose added to the medium. Cells were then frozen using a directional freezing device. The frozen samples were placed in a Labconco Freezone Plus 6 freeze dryer near the condenser for 3.5 days. The authors describe this freeze-drying system as “a very simple device, in which neither shelf temperature nor vacuum pressure can be controlled or recorded”. After processing, samples were stored at 2–8°C and 25°C for 1 week. The number of CD34+ cells were quantified by flow cytometric analysis after thawing frozen samples and rehydrating lyophilized samples. There were approximately 3.5±6×10^6^ and 5.3±12.3×10^6^ CD34+ cells after thawing and rehydration of lyophilized cells, respectively compared to fresh control (5.4±4.7×10^6^). These CD34+ cells produced 7.83±11.69 and 2.56±3.43 BFU-E following thawing and rehydration, respectively compared to control (14.67±15.89). The number of CFU-E produced by CD34+ cells were approximately 29.33±38.99 and 18.78±14.35 after thawing and rehydration, respectively compared to control (16.33±9.29). They did not report any observance of CFU-GEMM colonies.

The greater differentiation and clonogenic potential we observed when compared to that of Natan et al. may be directly related to the presence of intracellular trehalose. The results presented here show that transfer of trehalose into HPC was sufficient to maintain cell function during drying, storage at 25°C for 4 weeks, and reconstitution.

We propose that trehalose protects cells by the following mechanisms: (1) during cooling, ice nucleation occurs in the extracellular environment while the intracellular water supercools. Because of extracellular ice formation, solute concentrations are higher outside the cells than inside. As a result, water diffuses out of the cell. The cells dehydrate sufficiently such that intracellular ice formation does not occur. (2) As the temperature continues to decrease, the unfrozen solution within cells and the extracellular unfrozen fraction solidify into glass. The viscosity is sufficiently high to reduce molecular motion on a practical time scale. (3) The glass is maintained during desiccation and continues to protect cells during storage in the dried state. (4) Due to the relatively high amount of residual water, approximately 6.5%, for cells processed at 15°C, it would be unlikely that the water replacement hypothesis (see below) explains a mechanism of protection for cells processed at 15°C. In this case, the residual water could maintain hydration of biomolecules and serve to reduce damage during the drying and storage.

However, samples exposed to secondary drying at 20°C had approximately 1.8% residual water. For these cells in these samples, the water replacement hypothesis is a reasonable mechanism. The water replacement hypothesis suggests that adding trehalose before drying lowers the transition temperature (T_m_) of the dry membranes by replacing the water between the lipid headgroups, preventing the phase transition and its accompanying leakage upon rehydration[75], [76]. Desorption of water without trehalose increases the headgroup packing of phospholipid bilayers and forces the acyl chains together, increasing the probability of van der Waals interactions[77]. As a result, the lipids may undergo a transition from liquid crystalline to gel phase[75]. Upon rehydration, dry membranes, which are in gel phase at room temperature, undergo a transition from gel to liquid crystal phase. As the membranes pass through this phase transition there are regions with packing defects, making the membranes leaky; either due to membrane fusion or the induction of the hexagonal II phase[78]. In addition to lowering the T_m_ of membranes, trehalose and sucrose have been shown to preserve both structure and function of isolated proteins during drying[79], [80]. This ability to stabilize proteins during drying results from the disaccharides forming hydrogen bonds with the proteins when water is removed, thus preventing protein denaturation[81], [82], [83].

In conclusion, our results demonstrate that high levels of immature primary HPC derived from UCB can be recovered after lyophilization and ambient storage when permeabilized with the P2Z receptor and loaded with intracellular trehalose. Furthermore, improving the levels of mature HPC may be achieved by optimization of lyophilization processing conditions and the formulation. This methodology may be utilized with other cells expressing this pore forming protein. The P2Z receptor present in the brain is expressed by microglia and ependymal cells rather than neurons[58]. Since microglia are the resident macrophages of the brain[84], it is consistent with the finding of the P2Z receptor in peripheral macrophages and macrophage-like cells found in the lung and spleen[57]. Expression of this receptor has also been described in other tissues and systems. For example, primary human cells such as fibroblasts[85], fetal keratinocytes[86], vascular endothelial cells [87] and vein smooth muscle[88] have been permeabilized via P2Z receptor-associated pores. Cells derived from mice, such as medulla oblongata[89], parotid acinar cells[90], and spinal cord cells[89] along with immortal microglial[91], astrocytes [92], CHO-K1[91], and mesangial cells[93],[58] have also been permeabilized with ATP via the P2Z receptor. However, the most prominent expression is the hematopoietic tissue: dendritic cells[59], [94], duct cells[90], erythrocytes[95] and erythroid progenitors, granulocytes[58], macrophages[59], mast cells, monocytes[58], [96], neutrophils[64], peripheral blood mononuclear cells (CD34+)[97], promyelocytes[64], [98], and thymocytes[99] including B lymphocytes[85] and T lymphocytes[100]. With such a variety of cells already known to express this protein, there is potentially a wide application for freeze-drying cells with intracellular trehalose followed by ambient storage.
